# Tracing the dynamic life story of a Bronze Age Female

**DOI:** 10.1038/srep10431

**Published:** 2015-05-21

**Authors:** Karin Margarita Frei, Ulla Mannering, Kristian Kristiansen, Morten E. Allentoft, Andrew S. Wilson, Irene Skals, Silvana Tridico, Marie Louise Nosch, Eske Willerslev, Leon Clarke, Robert Frei

**Affiliations:** 1 National Museum of Denmark, Frederiksholms Kanal 12, DK-1220, Copenhagen K, Denmark; 2 Danish National Research Foundation’s Centre for Textile Research (CTR), SAXO Institute, University of Copenhagen, Amagerfaelledvej 56, Copenhagen 2300, Denmark; 3 Institute for Historical Studies, University of Gothenburg, Box 200, 405 30 Gothenburg, Sweden; 4 Centre for GeoGenetics, Natural History Museum of Denmark, University of Copenhagen, Øster Voldgade 5-7, Copenhagen 1350, Denmark; 5 School of Archaeological Sciences, University of Bradford, Bradford, West Yorkshire, BD7 1DP, United Kingdom; 6 School of Veterinary and Life Sciences, Murdoch University, Perth, Western Australia 6150, Australia; 7 School of Science and the Environment, Faculty of Science and Engineering, Chester Street, Manchester, M1 5GD, United Kingdom; 8 Department of Geoscience and Natural Resource Management, University of Copenhagen, Øster Voldgade 10, Copenhagen 1350, Denmark; 9 Nordic Centre for Earth Evolution (NordCEE), University of Copenhagen, Øster Voldgade 10, Copenhagen 1350, Denmark

## Abstract

Ancient human mobility at the individual level is conventionally studied by the diverse application of suitable techniques (e.g. aDNA, radiogenic strontium isotopes, as well as oxygen and lead isotopes) to either hard and/or soft tissues. However, the limited preservation of coexisting hard and soft human tissues hampers the possibilities of investigating high-resolution diachronic mobility periods in the life of a single individual. Here, we present the results of a multidisciplinary study of an exceptionally well preserved circa 3.400-year old Danish Bronze Age female find, known as the Egtved Girl. We applied biomolecular, biochemical and geochemical analyses to reconstruct her mobility and diet. We demonstrate that she originated from a place outside present day Denmark (the island of Bornholm excluded), and that she travelled back and forth over large distances during the final months of her life, while consuming a terrestrial diet with intervals of reduced protein intake. We also provide evidence that all her garments were made of non-locally produced wool. Our study advocates the huge potential of combining biomolecular and biogeochemical provenance tracer analyses to hard and soft tissues of a single ancient individual for the reconstruction of high-resolution human mobility.

Recent advances in tracing techniques at the individual level provide us with methodologies to map individual mobility during different life stages^1^,^2^,^3^,^4^,^5^,^6^,^7^,^8^,^9^,^10^,^11^,^12^,^13^,^14^. However, the limited preservation of coexisting hard and soft human tissues often impedes the diachronic investigation of a single individual.

Here we investigate the remarkable remains of the iconic Egtved Girl, who belongs to an impressive group of Bronze Age oak coffin burials from Denmark that were placed in monumental elite burial barrows dated to 1500-1100 BC^15^. Excavations in 1921, close to the village of Egtved in Denmark (Fig. 1), revealed the partially preserved remains of a high status, fully dressed female of approximately 16 to 18 years of age (Fig. 2). Dendrochronological analysis indicates that she was buried in an oak coffin approximately 3,400 years ago^16^,^17^. Hair, tooth enamel, nails, and parts of the brain and skin are still preserved, but no bones survived, most likely due to their dissolution in the partially acidic waterlogged environment prevailing within the oak coffin. A small container with some cremated skeletal remains of a 5 to 6-year-old child was placed by her head.

Her extremely well preserved costume consists of several textiles including a short corded skirt, a short blouse (Supplementary Table S1) and a disc-shaped bronze belt plate (Fig. 2) symbolizing the sun, which has been interpreted as belonging to a priestess of the Nordic sun worshipping cult^15^. Hence, the large variation of preserved hard and soft tissues, together with a rich assemblage of different grave goods provides a unique opportunity to investigate mobility at the individual level.

## Results

### Hard human tissues

**Figure 1:**
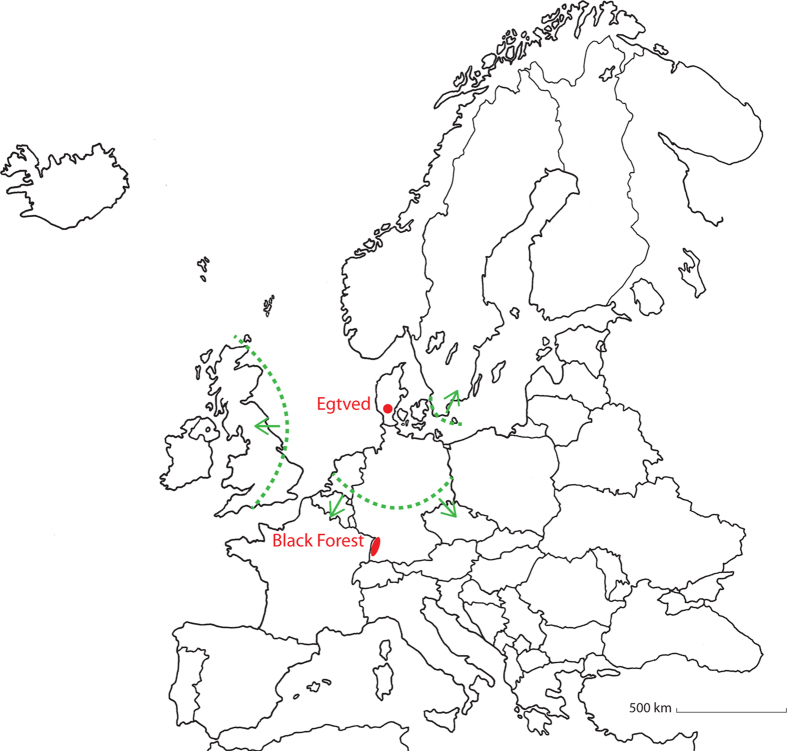
Map showing the location of the Egtved burial site (red dot). Borders of the nearest areas with bioavailable ^87^Sr/^86^Sr values that potentially fit the tooth enamel, the child’s bone, wool garments and oxhide belonging to the Egtved find are marked with green lines and arrows. Of these regions the Black Forest area (red ellipse) appears to be the most plausible place of origin as constrained by the multiple strontium isotope codes contained in materials from the Egtved find combined with the archaeological artefact record patterns. (Drawing by Marie Louise Andersson, with kind permission of the National Museum of Denmark).

To trace the Egtved Girl’s origin, we performed a strontium isotope analysis of enamel from the left mandibular first molar tooth. With the exception of the third molar, tooth enamel mineralizes during early childhood (e.g. the 1st molar mineralizes between peri-natal to 3 to 4 years of age) and it does not remodel thereafter, hence carrying childhood information on geographic origin^6^ (Supplementary Information). The Egtved Girl’s tooth enamel yielded a ^87^Sr/^86^Sr value of 0.71187 (± 0.0002; 2σ; Table 1). Similarly, we measured the strontium isotope signature of the associated child’s compact part of the occipital bone, the *pars petrosa* which was recently shown to be a valuable archive preserving origin information^5^ (Supplementary Information). The occipital bone yielded an ^87^Sr/^86^Sr value of 0.71190 (± 0.0002; 2σ; Table 1), a value which is indistinguishable from the Egtved Girl’s tooth enamel. Studies aimed at delineating the range of bioavailable strontium isotope compositions characteristic for present day Denmark (excluding the island of Bornholm and hereafter referred to as “Denmark”) resulted in a baseline range defined by ^87^Sr/^86^Sr values of 0.708 to 0.711^18^,^19^,^20^,^21^ (Supplementary Information). Bioavailable strontium from the Egtved burial site itself is defined by isotopic compositions which lie in the lower end of this scale with ^87^Sr/^86^Sr values ranging from 0.70852 to 0.70874 (Table 1). Hence, comparing the strontium isotope results from the Egtved Girl and accompanying child with the Danish baseline, it implies that both individuals originated from outside present day Denmark (Fig.1).

### Human soft tissues

**Figure 3:**
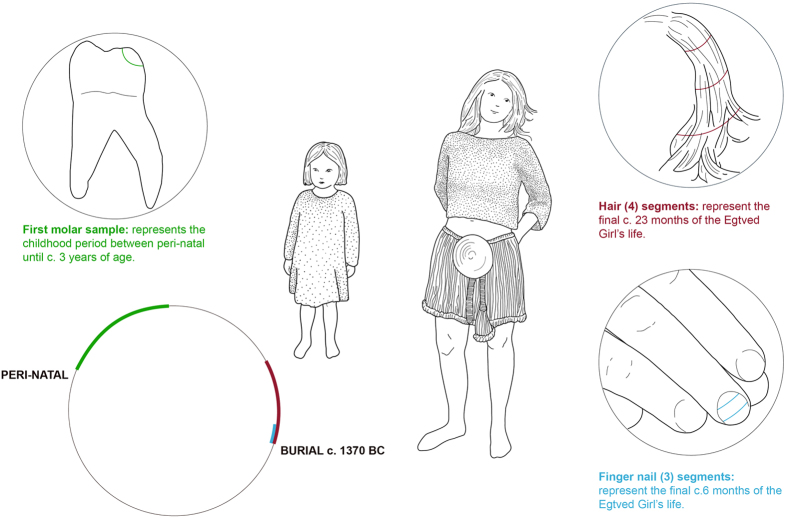
Drawing depicting the sampling strategy to reconstruct a high-resolution life-mobility-timeline of the Egtved Girl. We sampled tooth enamel to reconstruct the first years of her life, segments of scalp hair to reconstruct, at least, the 23 final months of her life as well as segments of one of her fingernails to reconstruct the final approximately 6 month of her life. (Drawing by Marie Louise Andersson, with kind permission of the National Museum of Denmark).

To trace the Egtved Girl’s mobility during the final months of her life, we divided her 23-cm long scalp hair into 4 segments covering a total growth period of, at least, 23 months prior to death (Fig. 3 and Supplementary Information). The oldest period represented by the hair (segment 4, Table 1) which corresponds to, at least, 23 to 13 months prior to death, is characterized by an elevated strontium isotope signature (^87^Sr/^86^Sr = 0.71255). The middle segments 2 and 3 represent a period of, at least, 9 months, and have similar lower strontium isotope signatures (^87^Sr/^86^Sr = 0.71028 to 0.71086). These two middle segment values are compatible with bioavailable signatures characteristic for Denmark^18^,^19^,^21^. However, the youngest scalp hair segment 1, corresponding to, at least, the final 4 to 6 months of the Egtved Girl’s life, again reveals an elevated strontium isotope signature (^87^Sr/^86^Sr = 0.71229) similar to that measured in the oldest part of the hair. Finally, data of the three segments from one of her fingernails (^87^Sr/^86^Sr = 0.71235 to 0.71240) corroborate with the youngest hair segment signature, which together cover the same final 4 to 6 months of her life.

Stable isotope signatures (*δ*^15^N = 8.6‰; *δ*^13^C = −21.6‰) of a c. 6-cm-long scalp hair corresponding to the same period as the youngest scalp hair segment analyzed for its strontium isotope composition indicate a terrestrial diet (Supplementary Table S3, Supplementary Fig. S3 and Supplementary Information). The partial sigmoidal curve defined by *δ*^13^C and *δ*^15^N could be suggestive of a seasonal diet variation, although the variance in nitrogen isotopes too, may potentially be interpreted as resulting from physiologically-related influences (Supplementary Fig. S3). Additionally, micro-morphological investigations of several scalp hairs reveal marked constrictions along shafts which may reflect periods of reduction/availability of protein^22^ (Supplementary Fig. S5 and Supplementary Information).

DNA was extracted from the Egtved Girl’s scalp hair, and using high throughput sequencing technology we obtained >28 million DNA sequences (Supplementary Table S2 and Supplementary Fig. S2) which we intended to use for elucidating population affinity and phenotypic characters (Supplementary Information). However, the proportion of non-duplicated sequences identified as human was extremely small (0.04% and 0.13%, respectively in two extracts) and did not exhibit the molecular characteristics expected for ancient DNA with increased levels of cytosine deamination damage towards the termini. We therefore conclude that there is minor, if any, retrievable ancient human DNA preserved in the hair sample, most likely due to the acidic pH of the burial environment, combined with years of exhibition^23^.

### Garment fibres

The measurements of the wool fibres indicate extensive selection and processing (Supplementary Fig. S6, Supplementary Table S4 and Supplementary Information), indicative of high quality textiles. The strontium isotope compositions from the animal fibres from the textiles and the underlying oxhide reveal a large range from ^87^Sr/^86^Sr = 0.71168 to 0.71551 (Table 1, Supplementary Table S1 and Supplementary Information), revealing that her outfit was made of raw materials gathered from outside Denmark. Only the raw materials from a wool cord placed in the container with the cremated remains of the child (ad 11847a, Table 1) yielded strontium isotope signatures that could imply local origin (^87^Sr/^86^Sr = 0.70982 to 0.71044).

## Discussion

To map the mobility patterns of the Egtved Girl we compare our strontium isotope results with strontium isoscapes from areas adjacent to Denmark^21^,^24^,^25^,^26^,^27^,^28^,^29^,^30^ and combine this information with the archaeological record. As depicted in Fig. 1, several target areas/regions are potential provenance candidates (i.e. with ^87^Sr/^86^Sr values >0.711), however, the nearest areas lie several hundred kilometres away from the burial site. Furthermore, given the wide range of strontium isotopic compositions from this study (especially obvious in the textiles, Table 1), we focus on regions with a geological background that accounts for these variations. The archaeological record from this period reveals distribution patterns of artefacts spreading from areas in southern Germany towards the north into southern Scandinavia (Supplementary Fig. S1), and suggests inter-chief alliances through intermarriage with elite foreign women^15^. Southwestern Germany, and in particular the Black Forest (Fig. 1) and adjacent areas, are characterized by a Palaeozoic granite-gneiss core overlain by Triassic sediments with bioavailable strontium isotope signatures that overlap with the wide range of values presented here^26^. Thus, with the support of archaeological evidence, we propose, that the Egtved Girl, the child, and the garments, may all originate from an area around the Black Forest in southwestern Germany. However, the strontium isotope results could also match other parts of Europe with similar isotopic compositions of bioavailable strontium (Fig. 1)^21^,^24^,^25^,^26^,^27^,^28^,^29^,^30^. Regardless of the difficulties in determining her exact provenance, our results point to a high level of mobility especially during the last two years of the Egtved Girl’s life. During this period she consumed a terrestrial diet but experienced periods of poor protein intake while she was moving back and forth from a place outside Denmark to an area characterized by bioavailable strontium with a less radiogenic signature, such as typical of northern Germany, and Denmark. The strontium isotopic signatures in her fingernail and most recent hair segment imply that she traveled from a place distant to Egtved shortly prior to her death.

Our study provides evidence for long-distance and periodically rapid mobility. Our findings compel us to rethink European Bronze Age mobility as highly dynamic, where individuals moved quickly, over long distances in relatively brief periods of time.

## Methods

We conducted a multidisciplinary study of the various hard and soft human tissues, as well as well-preserved wool textile and oxhide fibres from the Bronze Age grave find of the Egtved Girl from Denmark, and applied state-of-the-art biomolecular, biochemical and geochemical analyses and techniques to reconstruct high-resolution mobility patterns and diet.

Pre-analytical cleaning and extraction of strontium from the tooth enamel, the *pars petrosa*, fingernail segments, scalp hair, wool textiles fibres, and oxhide hair fibres followed appropriate procedures according to tissue type (see Supplementary Information for detailed descriptions)^1^,^2^,^3^,^5^,^6^,^8^. Thermal ionization mass spectrometry was used to determine the Sr isotope ratios. Samples were dissolved in 2.5 μl of a Ta_2_O_5_-H_3_PO_4_-HF activator solution and directly loaded onto previously outgassed 99.98% single rhenium filaments. Samples were measured at 1250-1300 °C in dynamic multi-collection mode on a VG Sector 54 IT mass spectrometer equipped with eight faraday detectors (Institute of Geoscience and Natural Resource Management, University of Copenhagen). Five nanogram loads of the NBS 987 Sr standard yielded ^87^Sr/^86^Sr = 0.710236 +/−0.000010 (n = 10, 2σ).

Stable isotope (*δ*^15^N and *δ*^13^C) analyses of the human scalp hair samples were prepared according to standard protocols at the University of Bradford stable light isotope facility. Adherent soil and exogenous organic deposits were removed (see Supplementary Information) and fibres were carefully orientated and aligned relative to the proximal end and to one another, so that fibre segments could be weighed into tin capsules and analyzed for a diachronic picture of change. Prepared hair samples were combusted in a Europa Scientific Geo 20/20 isotope ratio mass spectrometer coupled to a Roboprep elemental analyzer. Isotopic concentrations for each element are expressed in relation to international standards so that the relative difference between the sample isotope ratio and that of the standard is expressed by use of the *δ* notation, with units expressed as per mil (‰). Carbon is measured relative to CO_2_ prepared from a Cretaceous belemnite, from the Peedee Formation, South Carolina, whereas atmospheric N_2_ is used as the standard for nitrogen.

DNA was extracted utilizing phenol-chloroform combined with MinElute columns (Qiagen) as described previously^7^. Following extraction, 20 μl of DNA extract was built into a blunt-end library using the NEBNext DNA Sample Prep Master Mix Set 2 (E6070) and Illumina specific adapters (details in Supplementary Information). The DNA libraries were profiled on an Agilent Bioanalyzer 2100, pooled with other indexed libraries (different projects), and shot-gun sequenced (100 bp, single read) in two different sequencing runs on Illumina HiSeq 2000 platforms at the National High Throughput DNA Sequencing Centre, University of Copenhagen. The sequences were base called and sorted bioinformatically by index. Adapter sequences were trimmed off and reads shorter than 30 bp were removed using AdapterRemoval v.1.5.2^31^. Mapping against the human reference genome (hg19, build 37) was conducted with BWA v. 0.7.5^32^ with seeding disabled (-l 1000). Duplicates were removed from the bam file using SAMtools v. 0.1.18^33^ and only reads with mapping quality ≥25 were retained. See Supplementary Information for further details of DNA extraction, library build, amplification, sequencing and bioinformatics. Morphological investigations of scalp hair from the Egtved Girl were made by mounting hair fibres in a permanent mounting medium (Safe-T-Mount; R.I. 1.52) on conventional glass microscope slides and 0.17 mm thick cover slips. Microscopic investigations were performed with an Olympus compound transmitted light microscope, equipped with objectives ranging from 40-400x magnification.

Microscopic investigations and measurements of the diameter of the individual wool fibres sampled from the textile yarns were performed with a Zeiss Primo Star iLed transmitted light microscope with objectives ranging from 40-400x magnification, and images were captured with an AxioCam ERc5s digital camera. Fibre samples were mounted in liquid paraffin between conventional glass microscope slides and cover slips. A minimum of 100 fibres from each yarn sample were measured for their thicknesses on the photographs using the camera software.

## Author Contributions

Author KMF initiated the multidisciplinarity of the project. K.M.F., R.F., UM, M.E.A., S.T., A.W., K.K., I.S., M.L.N. and E.W. were responsible for writing the main manuscript text. KMF, MEA, AW, IS, ST and RF were responsible for conducting and interpreting the data, UM and KK were responsible for the archaeological context. KMF and RF performed all the strontium isotope analyses (scalp hair, tooth enamel, cremated bone, fingernail, wool textile fibres, soil and water), IS performed the microscopy investigations of the wool fibres and of scalp hair MEA performed the DNA analyses, AW and LC performed the stable isotope analyses and ST performed the morphological investigations. All authors reviewed the manuscript.

## Additional Information

**How to cite this article**: Frei, K.M. *et al.* Tracing the dynamic life story of a Bronze Age Female. *Sci. Rep.* **5**, 10431; doi: 10.1038/srep10431 (2015).

## Supplementary Material

Supplementary Information

## Figures and Tables

**Figure 2:**
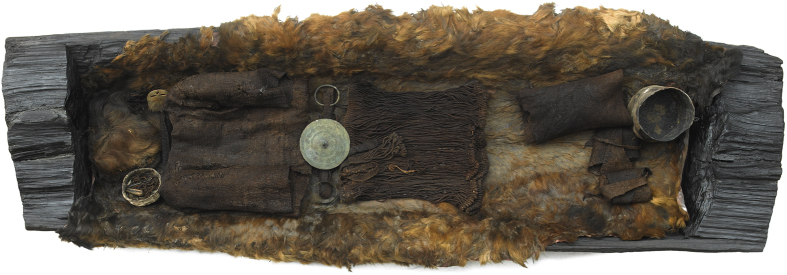
A photo of the remains of a Bronze Age high status female found inside an oak-coffin in a monumental burial barrow at Egtved, Denmark. The Egtved Girl’s garments are extremely well preserved and her exceptional wool costume consists of several wool textile pieces as well as a disc-shaped bronze belt plate, symbolizing the sun. (Photo: Roberto Fortuna, with kind permission of the National Museum of Denmark).

**Table 1:** Strontium isotope results from human remains of the Egtved Girl (tooth, hair and nail), textiles, oxhide, child bone and soil leachates.

**Sample**	**Sample description**	**material**	^**87**^**Sr/**^**86**^**Sr**	**2 SE**
***Local soils from the Egtved burial site (local baseline)***				
1	Soil from Egtved	Soil leachate	0.70852	0.00005
2	Soil from Egtved	Soil leachate	0.70874	0.00002

***Human remains (Egtved Girl and child)***				
ad B11831-50	Tooth, M1	Tooth enamel	0.71187	0.00002
ad B11821-50	Nail, thumb left side, oldest part	Nail	0.71235	0.00003
ad B11821-50	Nail, thumb left side, middle part	Nail	0.71240	0.00002
ad B11821-50	Nail, thumb left side, youngest part	Nail	0.71235	0.00003
11850 (segment 1)	Scalp hair, nearest the roots/skull, 4 cm long	Scalp hair	0.71229	0.00002
11850 (segment 2)	Scalp hair, middle part, 4 cm long	Scalp hair	0.71028	0.00003
11850 (segment 3)	Scalp hair, middle part, 5 cm long	Scalp hair	0.71086	0.00004
11850 (segment 4)	Scalp hair, tip ends, 10 cm long	Scalp hair	0.71255	0.00003
	Cremated remains of child	*pars petrosa*	0.71190	0.00002

***Textile and oxhide samples from within the oak coffin***				
B11834	Blouse (warp)	Wool	0.71234	0.00003
B11836	Corded skirt (weft)	Wool	0.71168	0.00003
B11849	Bundle (weft)	Wool	0.71551	0.00004
B11835	Belt (weft)	Wool	0.71277	0.00004
B11838	Foot wrapper, left foot (warp)	Wool	0.71319	0.00004
B11839	Foot wrapper, right foot (warp)	Wool	0.71530	0.00003
B11846	Oxhide hair	Hair/Fur	0.71324	0.00004
B11833	Blanket (light weft)	Wool	0.71252	0.00005
B11833	Blanket (dark weft)	Wool	0.71372	0.00003
B11833	Blanket (warp)	Wool	0.71399	0.00003
ad 11847a	Oxtail hair attached to wool cord	Oxtail Hair	0.70982	0.00002
ad 11847a	Oxtail hair detached from wool cord	Oxtail Hair	0.71003	0.00003
ad 11847a	Wool cord	Wool	0.71044	0.00004
