# Supplementary material for: Tracing the dynamic life story of a Bronze Age Female
Source: Sci Rep. 2015 May 21;5:10431. doi: 10.1038/srep10431 (PMC4440039; doi:10.1038/srep10431)
Supplement: Supplementary Information [file srep10431-s1.pdf]

Supplementary Information for:

Tracing the dynamic life story of a Bronze Age Female

Karin Margarita Frei [1,2] * Ulla Mannering [1, 2] Kristian Kristiansen[3], Morten E. Allentoft [4], Andrew S. Wilson[5], Irene Skals[1], Silvana Tridico[6], Marie-Louise Nosch[2], Eske Willerslev[4], Leon Clarke[7] and Robert Frei[8,9]

[1]National Museum of Denmark, Frederiksholms Kanal 12, DK-1220, Copenhagen K, Denmark

[2]Danish National Research Foundation's Centre for Textile Research (CTR), SAXO Institute, University of Copenhagen, Amagerfaelledvej 56, Copenhagen 2300, Denmark

[3]Institute for Historical Studies, University of Gothenburg, Box 200, 405 30 Gothenburg, Sweden

[4]Centre for GeoGenetics, Natural History Museum of Denmark, University of Copenhagen, Øster Voldgade 5-7, Copenhagen 1350, Denmark

[5]School of Life Sciences, University of Bradford, West Yorkshire, BD7 1D, Bradford, United Kingdom

[6]School of Veterinary and Life Sciences, Murdoch University, Perth, Western Australia 6150, Australia

[7]School of Science and the Environment, Manchester Metropolitan University, All Saints Building, All Saints, Manchester, M15 6BH, United Kingdom

[8]Institute of Geoscience and Natural Resource Management, University of Copenhagen, Øster Voldgade 10, Copenhagen 1350, Denmark

[9]Nordic Centre for Earth Evolution (NordCEE), University of Copenhagen, University of Copenhagen, Øster Voldgade 10, Copenhagen 1350, Denmark

*Correspondence to: Karin.M.Frei@natmus.dk

Archaeological context:

The Bronze Age marks a key transition in society giving rise to the beginning of state formation and urbanization which began in the Near East circa 3000 BC, and completed its early stages in central and northern Europe circa 1000-500 BC[15]. It was a period characterized by transformation and the transmission of know-how by long distance connections and alliances among local elites[15]. In Denmark, the oak-coffin graves covered by monumental mounds in which the elite were buried provide some information on their life and times. One of these oak-coffin graves is of a high-status-female, known as the Egtved Girl. She was buried approximately 3400 years ago on a summer's day as revealed by the flowers placed in her grave and by dendro-chronological analysis of the coffin[17]. Analyses of her teeth indicate she was approximately 16-18 years old when she died[17]. Of the girl herself, hair, teeth, nails and parts of the brain and skin remain (Fig.2). A small box made of birch bark was placed beside her head. This contained a bronze awl, the remains of a hair-net and parts of the cremated bones of a 5 to 6-year-old child[15].

The Egtved Girl is today an iconic figure from the European Bronze Age due to her extremely well preserved and exceptional costume consisting of several textile pieces, inter alia, a short blouse and a corded skirt (Supplementary Table S1 and Fig.2). Together with the disc-shaped bronze belt plate symbolizing the sun, her outfit has been interpreted as that of a priestess belonging to the Nordic sun-worshipping cult[15].

Supplementary Table S1. Description of costume and items found together with the Egtved Girl.

	Item	Museum number	Description	Function	Size	Strontium isotope analysis	No. of fibre analyses
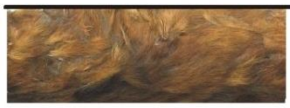	Ox hide	B11832	Primarily hairs preserved	Lining and cover		X	
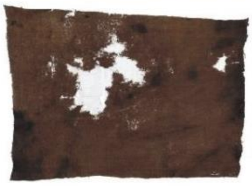	Blanket	B11833	Woven wool textile	Covering the body	170-192x250-258 cm	X	3
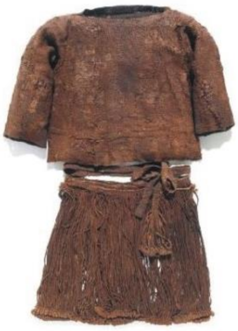	Blouse	B11834	Woven wool textile	Covering the upper body	104x44 cm	X	5
	Belt	B11835	Woven wool textile	Wrapped around the waist	162x2 cm	X	2
	Corded skirt	B11836	Twisted wool yarns	Covering the lower body	160x37 cm	X	7
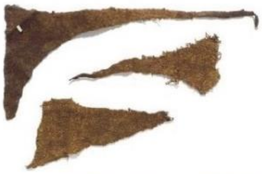	Left foot wraps	B11838	Woven wool textile	Covering the feet	50x25 and 25-50 cm	X	
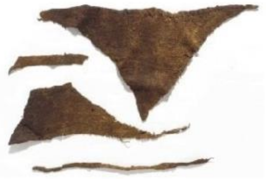	Right foot wraps	B11839	Woven wool textile	Covering the feet	30x11 and 14x26 cm	X	
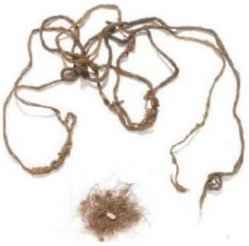	Cord	Ad B11847	Twisted wool yarns	Cord with hairs, possibly an amulet	245 cm	X	1
	Cow hair	Ad B11847a	Hairs worked into the cord			X	
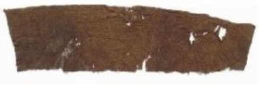	Textile	B11849	Woven wool textile	Wrap for the cremated bones	30x110 cm	X	2

The Egtved Girl is one of the very few female burials in an area dominated by male warrior graves. However, there is archaeological evidence for inter-chief alliances through intermarriage with elite women as shown in Supplementary Fig. S1[15].

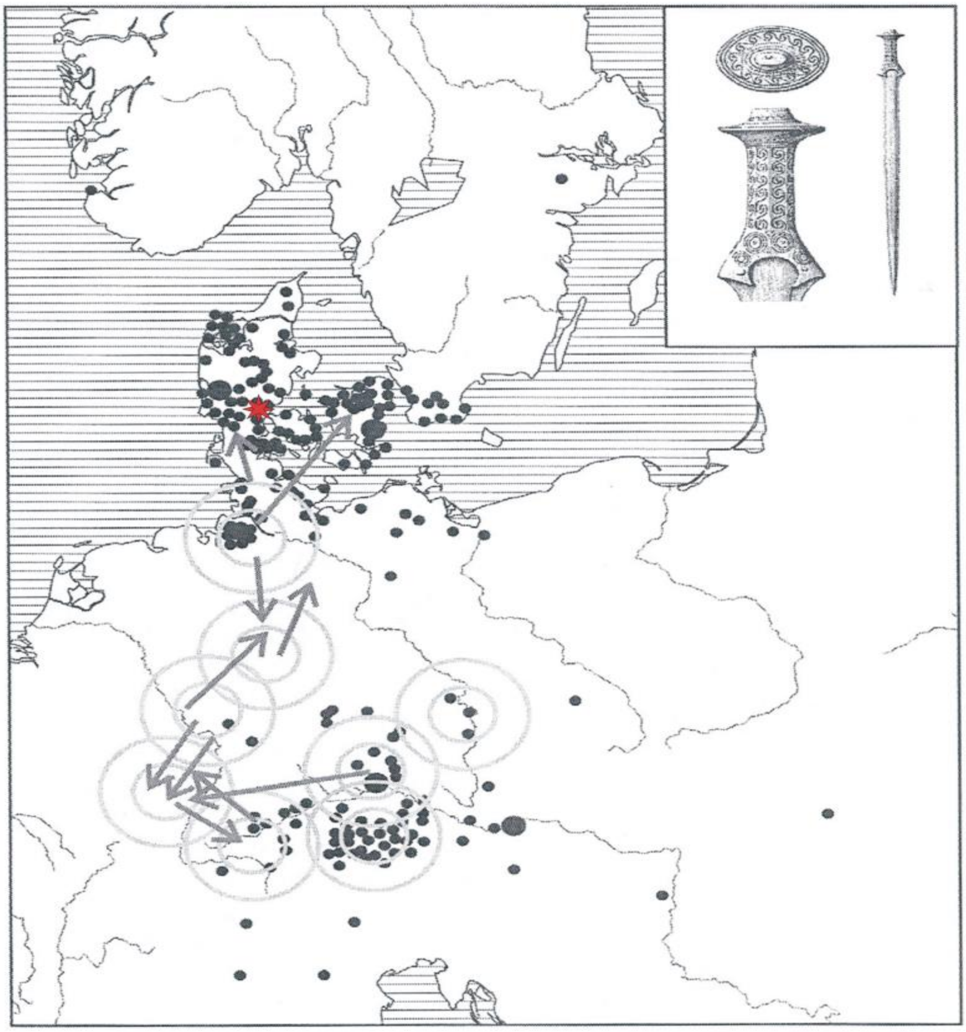

Supplementary Fig. S1. Map of Germany and Denmark depicting the patterns of inter-chief alliances during the Bronze Age, map modified after Kristiansen and Larsson [15]. The map shows the distribution of octagonal hilted swords (black dots) from the Bronze Age (Montelius period II) combined with homelands of local elite groups/polities (circles) and with intermarriage patterns with foreign women (arrows pointing to the respective women's origin). The red star marks the Egtved site.

We conducted a multidisciplinary study of the various human tissues, as well as her well- preserved wool garments, and applied state-of-the-art biomolecular, biochemical and geochemical analyses and techniques to reconstruct high-resolution mobility patterns and diet.

We conducted radiogenic strontium isotope tracing investigations of the Egtved Girl's scalp hair, teeth and nails representing different ontogenetic stages of her life, her exceptional and well-preserved wool textiles and the oxhide that lined the grave, as well as the cremated bones of a child buried together with her (Supplementary Table S1). We also applied state-of-the-art ancient DNA-, stable isotope (δ^{15}N and δ^{13}C) analyses as well as conducted microscopic investigations of the human scalp hair. Additionally, we performed microscopic investigations of the wool textiles. This is to our knowledge, the very first time that a systematic combination of provenance analyses has been conducted on hard and soft tissues of a single ancient individual to reconstruct high-resolution mobility.

<u>Strontium isotopes</u>

Human tooth enamel and cremated human bones of a child

Human tooth enamel from the first molar begins to form in utero and finalizes mineralization at c. three years of age[34] thus providing information of the place of childhood origin[6]. We therefore conducted a strontium isotope analysis of the first molar of the lower left jaw of the Egtved Girl. However, in order to obtain provenance information based on the strontium isotope system, baseline or isoscape ranges of the isotopic composition of local bioavailable strontium need to be known. Most of Denmark (hereafter defined as territorial Denmark) consists of a pre-Quaternary geological basement primarily composed of Tertiary and Cretaceous sediments (predominantly

carbonates), all characterized by relatively low strontium isotopic compositions, hence the baseline ranges from $^{87}Sr/^{86}Sr$ =0.708 to 0.711[14-16]. Only the island of Bornholm (located south of Sweden in the Baltic Sea) has elevated bioavailable strontium isotope signatures ($^{87}Sr/^{86}Sr > 0.711$) due to the Precambrian basement dominating most of the island [20] . Furthermore, we measured two soil leachates from samples collected at the Egtved burial site in order to characterize the local bioavailable strontium isotope range and obtained $^{87}Sr/^{86}Sr$ values of 0.70852 and 0.70874 (Table 1). For the above mentioned baseline measurements we followed the procedures described in Frei and Frei & Frei and Frei[18,20]. Our strontium isotope analysis of the (M1) tooth enamel of the Egtved Girl of $^{87}Sr/^{86}Sr = 0.71187$ (±0.0002; 2σ) implies, therefore, a non-local origin compared to the place of burial and to territorial Denmark.

We also conducted a strontium isotope analysis of the cremated human remains from the child buried together with the Egtved Girl. However, due the porosity of the bone structure there is a risk of post-depositional contamination as well as isotope fractionation during incineration, which could potentially alter the original signatures preserved in the skeletal tissues. Nevertheless, recent experimental investigations reveal that strontium isotope ratios remain unaltered even when exposed to high temperatures and that cremation processes result in an increase in crystallite size during recrystallization[35], which may even reduce diagenetic alteration processes. This would also explain the observation that the cremated bones have a tendency to be better preserved, something also reported from other archaeological finds in Denmark [17]. A recent study demonstrates that the compact part of the occipital bone, *pars petrosa*, is also a good archive from which information on the origin of a human individual can be deduced, as this bone forms during early childhood and does not remodel after the age of 2 [5]. We chose, therefore, to sample the *pars petrosa* from the assembly of cremated bones of the child buried with the Egtved Girl and measured an isotopic value of $^{87}Sr/^{86}Sr = 0.71190$ (±0.0002; 2σ) which we use to gain information on the child's

provenance. Our strontium isotope analysis of the the child's bone appears to also reveal a non-local provenance when compared to the baseline range measured from this site and from territorial Denmark as a whole. Interestingly, the tooth enamel from the Egtved Girl and the occipital bone of the child are characterized by statistically undistinguishable strontium isotope ratios, which confirms that the compact part of the bone was not affected by diagenesis and thus points to a likely shared place of origin for the Egtved Girl and child.

Human scalp hair, wool textiles and oxhide

Recent investigations show that a mild acid leaching of modern human hair with 0.1 M HCl is required to recover the strontium nutritional fraction of the hair necessary to investigate mobility [8]. Similar investigations of modern and ancient wool textiles demonstrate that a 20 % hydrofluoric acid (HF) leaching step effectively removes solid micro-particles as well as the lipid portion of hair known to be sensitive to environmental contamination [1-2]. We applied a combination of these methodologies to ensure the removal of the exogenous strontium portions of all mammal hair samples studied here, i.e. human, sheep/goat and cattle [1-3, 8].

Since modern human scalp hair grows c. 1 cm per month on average it is an archive which can potentially provide high-resolution diachronic information of diet and mobility when investigated by respective multi-disciplinary tracers [4,8, 9-11, 36-37]. Due to the archaeological nature of the find, the amount needed to perform reliable strontium isotope analyses impeded the preferred 1 cm segment sequencing. Hence, for the strontium isotope investigation of the hair we divided a 23-cm-long hair shaft into 4 segments covering a total growth period of, at least, 23 months prior to death. The oldest period represented by the hair (c. 23 -13 months prior to death) is characterized by an elevated strontium isotope signature ($^{87}Sr/^{86}Sr$ =0.71255). Both middle segments together represent a period of at least 9 months and have, however, lower strontium isotope signatures ($^{87}Sr/^{86}Sr$ =

0.71028 to 0.71086) which are compatible with bioavailable signatures in Denmark and northern Germany. The newest scalp hair segment representing, at least, the final 4 to 6 months of the Egtved Girl's life again reveals an elevated strontium isotope signature ($^{87}Sr/^{86}Sr$ = 0.71229) similar to the signature found in the end of the hair shaft. Finally, data ($^{87}Sr/^{86}Sr$ = 0.71235 to 0.71240) of the nail segments from one of her fingernails corroborates the youngest hair segment signature, which together cover, at least, the same final4 to 6 months period of her life. There is archaeological evidence for inter-chief alliances through marriage with elite women from elsewhere as demonstrated in Supplementary Fig. S1[15].

Recent developments in analytical protocols enables the extraction of the original strontium isotope fraction of wool textiles [1-3] in order to pinpoint the potential geographical areas where the sheep were grazing at the time that they were producing wool. Consequently, this permits the delineation of wool trade. In this particular case, we wish to assess the question of the Egtved Girl's mobility through life; hence her garments could potentially contribute to information on this issue. We conducted strontium isotope analysis of ten wool samples belonging to eight different textile pieces that the Egtved Girl was buried with. Additionally, we conducted strontium isotope analyses of hair from the oxhide fur and oxtail hair and wool from the remains of a wool cord found in the box beside her head.

The results from the wool fibres reveal a large range of strontium isotope compositions ranging from $^{87}Sr/^{86}Sr$ = 0.71044 to 0.71551. Surprisingly, only the wool cord sample (both wool and the attached oxtail hair, ad 11847a) have strontium isotope values that are compatible with values characterizing the baseline range typical for territorial Denmark (i.e. which ranges from $^{87}Sr/^{86}Sr$ =0.708 to 0.711). Hence, this is indicative of a possible Danish origin. However, all the other textiles (garments, belt and blanket) are made of non-local wool as they have $^{87}Sr/^{86}Sr > 0.711$

($^{87}Sr/^{86}Sr$ = 0.71168 – 0.71530). A non-local origin of the oxhide (B11846) is also indicated by its elevated strontium isotopic signature of $^{87}Sr/^{86}Sr$ = 0.71324. The wide range of strontium isotope compositions in the wool fibre samples analyzed here may indicate either early pastoralism or extended wool trade.

Assessing ancient DNA authenticity

To assess the authenticity of the DNA sequences that mapped to the human reference genome, we examined the data for two characteristics expected for ancient DNA: (1) A negative exponential correlation between the number of sequences and sequence length, caused by random fragmentation of the genome and (2) An increase in C to T changes (compared to the reference genome) in positions at read ends - an effect of post-mortem deamination of cytosine. Both these analyses were conducted with bammds[37].

Only small proportions (1.9% and 4.3%, Supplementary Table S2) of the sequences could be identified as human which is not unusual for shot-gun sequencing of ancient material. However, due to the low amount of DNA, the clonality is extreme (Supplementary Table S2). Hence, when subtracting identical reads, the proportions become 0.04% and 0.13% respectively (Supplementary Table S2). Moreover, owing to the lack of ancient DNA molecular characteristics in the two libraries (Supplementary Fig. S2) we conclude that the small amount of identified human DNA is unlikely to be ancient.

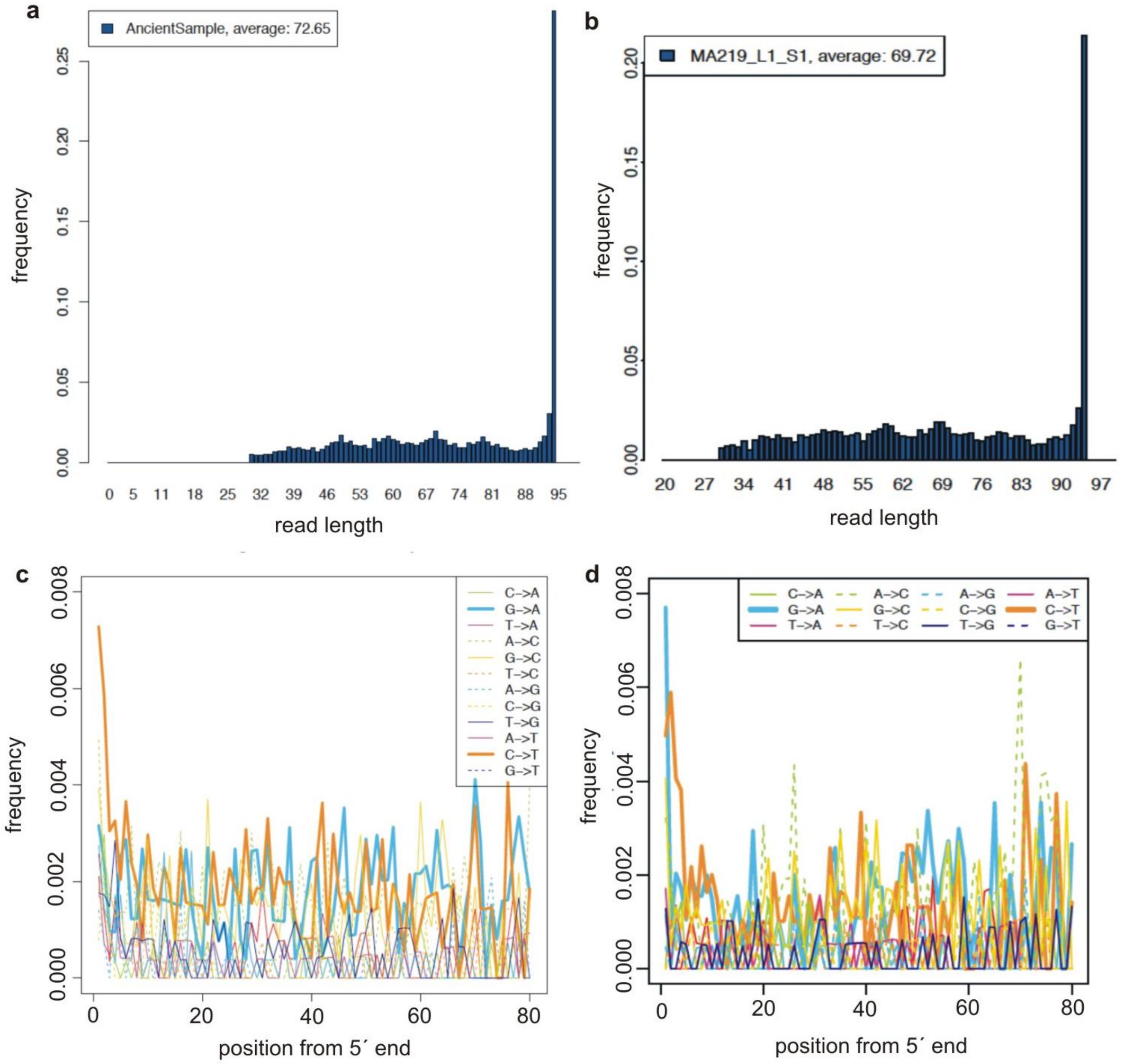

Supplementary Fig. S2. Molecular characteristics of the two DNA extracts from the Egtved Girl's hair. More than 28 million DNA reads were obtained but the profiles depicted here represent only those DNA sequences identified as human. Panels *a* and *c* are derived from extract MA218, and panels *b* and *d* are from MA219. The fragment length distributions do not display the negative exponential correlation as expected for degraded DNA51. Moreover, there is no increase, or only a very limited amount, of C to T DNA damage towards the 5' end of the sequences. This is a very strong indication that the few identified human DNA molecules are not of ancient origin.

Supplementary Table S2. Summary statistics of >28 million DNA sequences generated from two extracts of the Egtved Girl's hair. *Total*, represents the total number of sequences generated, *Mapped to Hg19* is the number of sequences (without duplicates) that could be mapped to the human reference genome 19, and *Human %* is the fraction of identified human sequences in the data.

Extract	Total post trim	Mapped Hg19 Dupl. removed	human % non dup.	Av. length
MA218	27,605,264	11,345	0.04 %	71.6 bp
MA219	616,489	818	0.13 %	69.6 bp

Stable isotopes

Isotopic data from hair will vary predictably depending on dietary intake [36-37, 39]and can, therefore, be used to distinguish between reliance on largely terrestrial-based or marine food resources in past populations, seasonal variation in diet and potentially identify physiological impacts. Carbon stable isotope ratios (δ^{13}C) and nitrogen stable isotope ratios (δ^{15}N) reflect the main sources of protein consumed at the time that the tissue forms. Nitrogen (δ^{15}N) values increase as a result of metabolic fractionation by 2-5 ‰ at each trophic level of a food chain and can, therefore, provide information on the relative consumption of plant, animal and marine protein where sufficient baseline data exists [40]. In the case of the Egtved Girl we were also able to undertake a bulk analysis of the oxhide hair associated with her.

In northwest Europe, carbon isotope values can be used to help distinguish the extent to which different plant and marine sources contribute to the diet, and in the case of serial measurements using hair segments, it is possible to determine the extent to which seasonality and other factors are broadly noted with time. Nitrogen isotopes can offer insight into dietary (protein), with δ^{15}N values increasing as the food chain is ascended. For instance a more carnivorous diet will therefore result

in higher δ^{15}N values than the herbivores on which they feed. Nitrogen isotopes can also be interpreted in the context of other physiological markers. Data on international and laboratory standards run alongside the samples for consistency and quality assurance purposes showed that the analytical error was determined at ±0.2‰ or better. Data are tabulated as either bulk measurements of δ^{15}N and δ^{13}C (Supplementary Table S3) or as separate plots showing incremental changes in the isotopic data (Supplementary Fig. S3).

Supplementary Table S3. Stable isotope data of bulk hair samples.

Sample	δ^{15}N (‰)	δ^{13}C (‰)	C:N
Egtved Girl hair segment a	8.4	-21.2	3.7
Egtved Girl hair segment b	8.7	-21.6	3.9
Egtved Girl hair segment c	8.8	-21.7	3.8
Egtved Girl hair segment d	8.5	-21.7	3.8
Mean of hair from 3 other Bronze Age oak coffin burials	9.2	-21.3	3.6
Mean Egtved Girl hair segments	8.6	-21.6	3.8
Mean Egtved ox-hide	3.9	-24.5	4.2

The bulk stable isotope data (δ15N = 8.6‰; δ13C = -21.6‰) for the Egtved Girl indicate a terrestrial diet, broadly consistent with mean values for hair from three other Danish Bronze Age oak-coffin burials (δ15N = 9.2‰; δ13C = -21.3‰) and contrasted with the lower trophic level (herbivorous) animal hair (δ15N = 3.9‰; δ13C = -24.5‰) found with her (Supplementary Table S3 and Supplementary Fig. S3). The variation in stable carbon and nitrogen values was very small, in

some cases being within the analytical error determined from repeat measurements of internal and international standards which was ± 0.2 ‰ or better. That makes trend data from one individual over such a short timescale (~6 months), difficult to interpret. Certainly, incremental measurements producing a sigmoidal curve for δ^{13}C can suggest seasonal variation in dietary intake. Trend data for nitrogen isotopes which show very limited variation should not be over interpreted, particularly since elevated nitrogen can potentially be due to a variety of contrasting factors, ranging from increased consumption of dietary protein, to nitrogen imbalance due to physiological stress – inclusive of illness, pregnancy or nutritional stress [39-42].

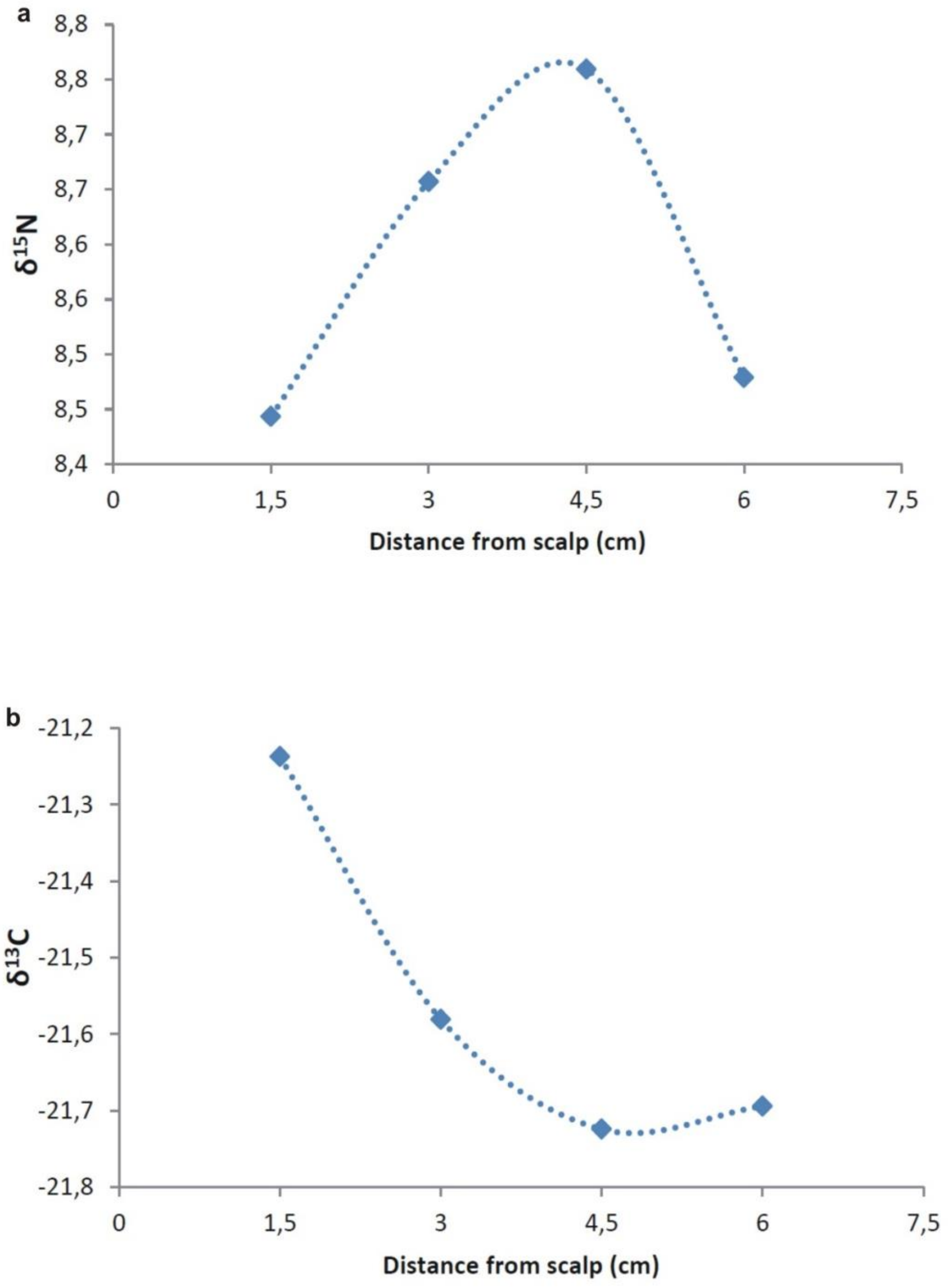

Supplementary Fig. S3. Bulk stable distribution pattern in scalp hair segments of the Egtved Girl. *δ*15N and *δ*13C analyses cover a period of at least 6 months prior to her death. The sigmoidal curve for *δ*13C is highly suggestive of seasonal variation in dietary intake.

Morphological investigations of scalp hair from the Egtved Girl

Preliminary examinations of the scalp hairs from the Egtved Girl (using visual inspection and low power microscopy under 6- 40x magnification) revealed a range of hair colours from dark red/brown, to black and pale to medium brown. In profile, the hairs are essentially straight (i.e. with minimal curl). The sample examined showed that the hairs are fragments, i.e. they lack roots, and measure between 50-105 mm in length. A number of hairs were selected for more detailed microscopic examination.

Our microscopical investigation reveals that a partially sheared hair shaft lacks visible pigment granules in the cortex and the presence of *cortical fusi* (air filled vacuoles) throughout the entire length of the hair shaft (Supplementary Fig. S4). An obvious lack of pigmentation and the overall pale brown coloration of many hairs support the premise that the Egtved Girl had natural blonde or light brown hair. Several scalp hairs of the Egtved Girl exhibit marked constrictions along shafts comparable to those exhibited by scalp hair from an individual of the Saqqaq culture [7]. These constrictions may reflect periods of reduction/availability of protein containing food [41] (Supplementary Fig. S5). However, a number of other causalities, including physiological stress, could potentially also cause these constrictions. The oval shape and dimensions of the transverse cross-section are typical for Caucasian scalp hairs [43-44].

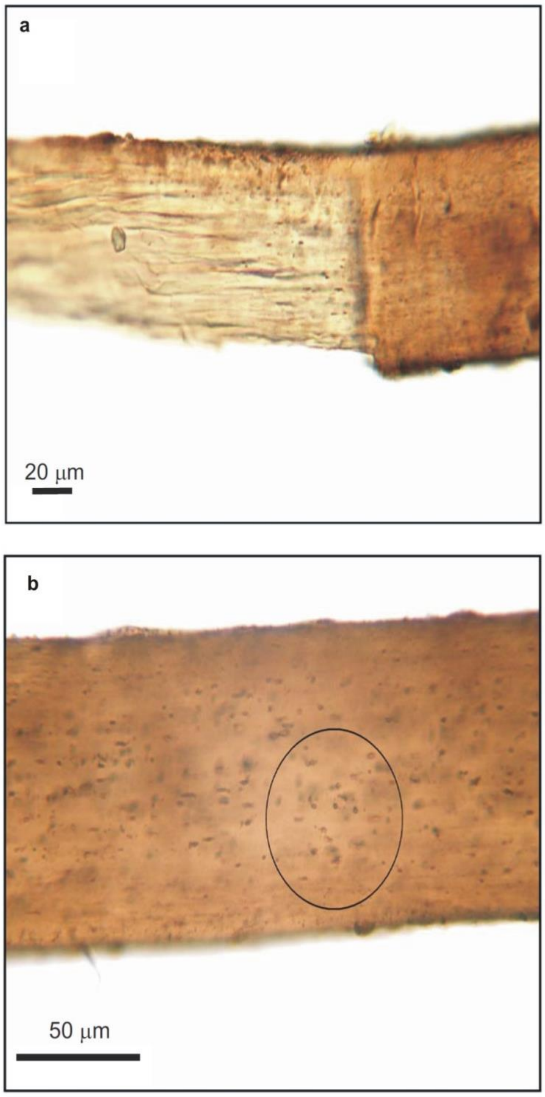

Supplementary Fig. S4. Photomicrograph of scalp hair of the Egtved Girl. Panel *a* shows partially sheared hair shaft revealing the cortex devoid of pigment granules. Panel *b* shows the cortical fusi present along the length of hair shafts and pigment granules (ellipse).

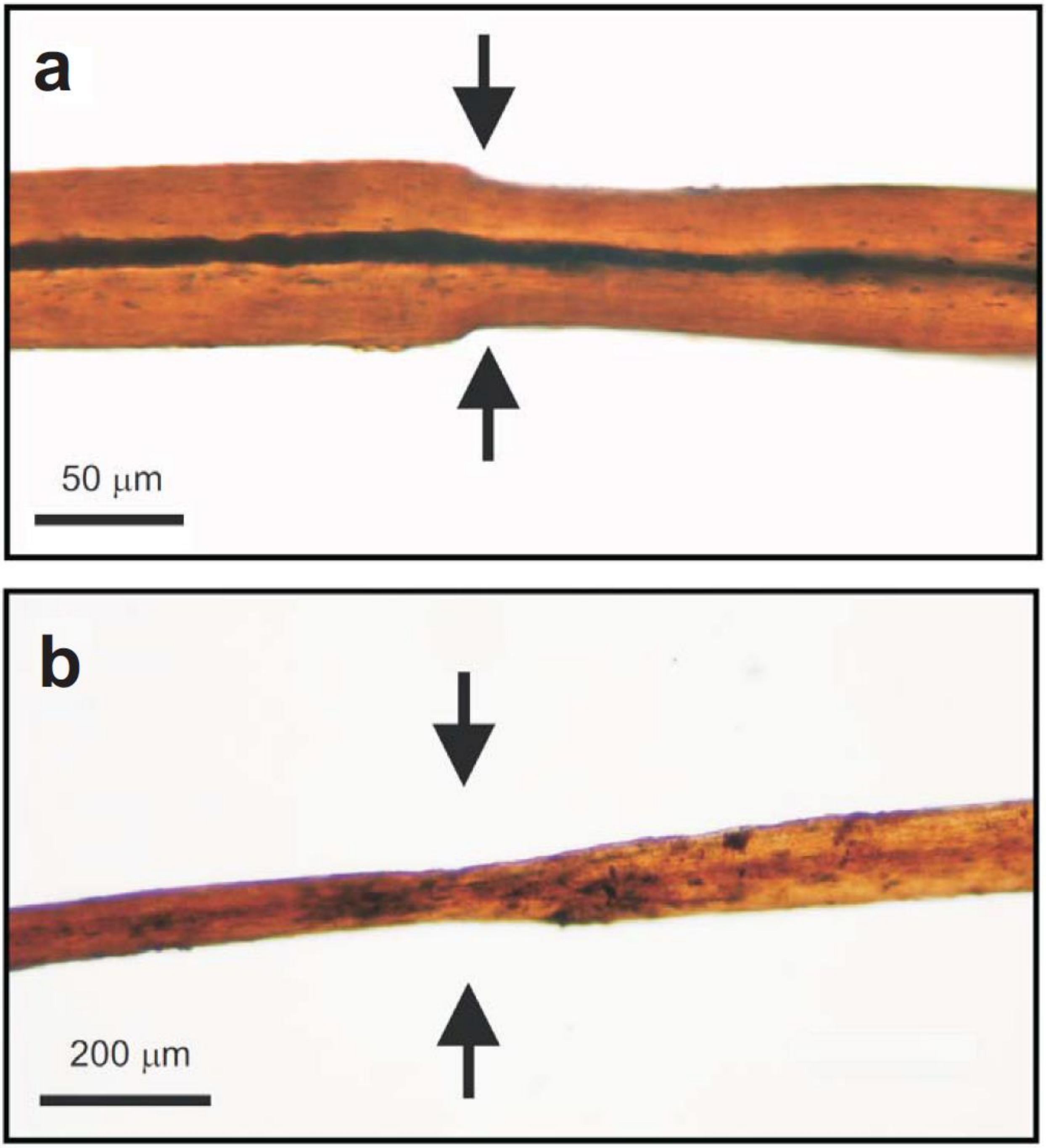

Supplementary Fig. S5. Photomicrograph of hair shafts from the Egtved Girl and a Paleo-Eskimo. Panel *a* shows constrictions (pinpointed by arrows) present in scalp hair taken from a male Paleo-Eskimo. The dark central band is the medulla (vacuolated structure present in many mammalian hairs). Panel *b* shows comparable constrictions in a hair shaft from the Egtved Girl.

Microscopy of wool fibres

One of the main criteria used to determine modern wool qualities is fibre dimensions defined by the fibre diameter, and similar criteria have also been applied to prehistoric wool textiles [45-46]. The methodology consists of measurements of the diameter of the individual wool fibres sampled from the textile yarns combined with statistical analyses of the measurements often depicted as histograms which enables the comparison between different samples and materials from different locations [6]. This standard analysis of wool fibre fineness is used to determine the fleece types of prehistoric sheep, enabling comparisons with fleece from modern sheep and leading to conclusions on ancient breeds [5]. A fleece consists of the outer coat containing coarse kemp (over 100 µm in diameter) and hair (over 60 µm in diameter), and the much finer underwool. Results from analyses of the wool in Scandinavian prehistoric textiles are interpreted as stemming from a fleece type containing a high amount of very fine fibres and an unknown amount of very coarse hairs and kemp [45, 47]. In a recent study of prehistoric wool textiles and fleeces from Norway, Sweden, Austria and the Balkans similar characteristics were identified among the North European Bronze Age textiles finds, leading to the conclusion that several different sheep breeds existed during the European Bronze Age and that North European sheep breeds, and thus their wool fibre composition, may have differed from those of Central Europe [46].

We measured wool fibres from the same yarn samples that were also analyzed for strontium isotopes (Supplementary Fig. S6). Altogether, 20 samples from these six costume items were included in the fibre analysis. The results of the microscopy investigations depict a narrow range in thicknesses indicating that a similar raw material was used for all the tested yarns. The differences in the amount of outliers (thick fibres) indicate small differences in the processing of the raw material from fleece to yarn. Although the present analysis was performed using transmitted light

microscopy versus SEM microscopy as in previous studies [46] our results are similar to those from other Northern European Bronze Age textiles. In comparison to the strontium isotope analysis of the wool fibres, the finished costume items show that the assemblages of garments were gathered from geologically diverse areas, testifying to a complex circulation of either wool as raw material or finished garments.

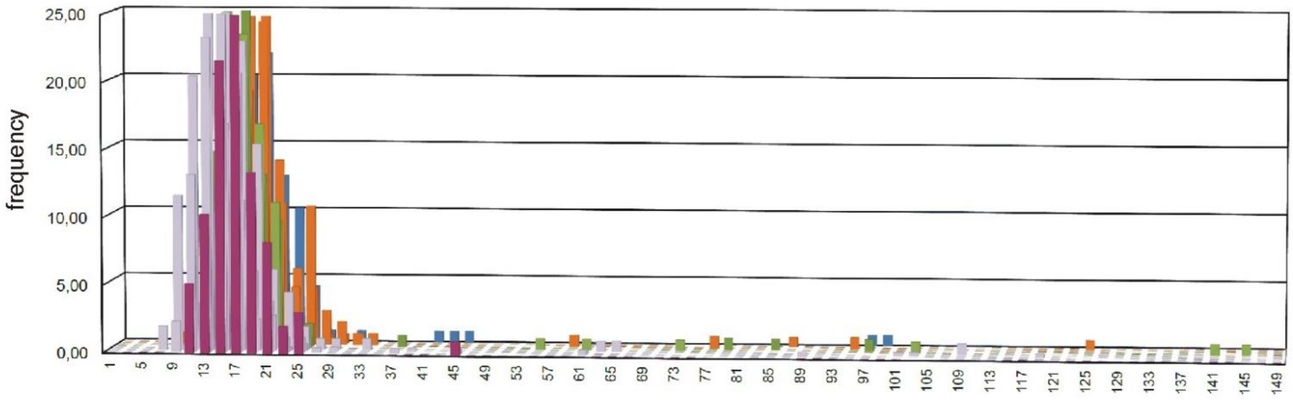

Supplementary Fig. S6. Distribution of wool fibre thicknesses from the Egtved Girl's garments. The histogram shows the frequency distribution of the fibre thicknesses (µm) of a single thread (separately coloured) from which an aliquot was analyzed for strontium isotopes (Table 1). The fibre thicknesses cluster in a narrow range (with few outliers) indicating that the different textiles from Egtved were made with similarly fine processed raw materials.

Methods

Strontium isotopes

Human tooth enamel and a fingernail from the Egtved Girl and cremated human bone from the accompanying child

The tooth enamel sample from the Egtved Girl was mechanically pre-cleaned with a dental drill and subsequently repeatedly washed ultrasonically in ultrapure (MilliQ™) water until the water remained visually clear. The petrous bone sample from the cremated remains of the child was leached with 1M acetic acid for 10 minutes. The thumb-nail was repeatedly washed ultrasonically in ultrapure (MilliQ™) water until the water remained visually clear. The cleaned residue of the fingernail was cut into three pieces perpendicular to the growth direction.

The clean enamel, bone and fingernail samples were placed in a 7ml Teflon beaker (Savillex™) and subsequently dissolved in a 1:1 mixture of 0.5 ml 6 N HCl (Seastar) and 0.5 ml 30% H_2O_2 (Seastar). The samples decomposed rapidly within a few minutes, after which the solutions were dried down on a hotplate at 80 °C.

The samples were taken up in a few drops of 3N HNO_3 and then loaded onto disposable pipette extraction columns with a 0.2 ml stem volume charged with intensively pre-cleaned mesh 50-100 SrSpec™ (Eichrome Inc.) resin. The elution recipe essentially followed that by Horwitz et al., [48]scaled to our needs. Strontium was eluted / stripped by pure deionized water and then the elute was dried on a hotplate. The strontium samples were dissolved in 2.5 µl of a Ta_2O_5-H_3PO_4-HF activator solution and directly loaded onto previously outgassed 99.98% single rhenium filament. The samples were measured at 1250-1300 °C in a dynamic multi-collection mode on a VG Sector 54 IT mass spectrometer equipped with eight Faraday detectors (Institute of Geoscience and Natural Resource Management, University of Copenhagen). Five ng loads of the NBS 987 Sr standard gave $^{87}Sr/^{86}Sr = 0.710236 \pm 0.000010$ (n = 10, 2 σ).

Human scalp hair, wool textiles and oxhide

The human hair samples, wool fibres and the animal hair fibres from the oxhide were all washed and cleaned in several acid steps. The first consisted of applying 1 ml of a 1N hydrochloric (HCl) acid to the sample for one hour in a 7ml Teflon beaker (Savillex™) in an ultrasonic bath. The acid was pippeted off and three rinsing steps of deionised water (MilliQ™) followed. The second step consisted of adding a 1:1 mixture of 20 % HF and deionised water (MilliQ™) to the residue. The solution was removed and the residue was rinsed in three times with deionized water (MilliQ™). Following the decontamination procedures, the hair residues (human, textile and fur) were dissolved in a 1:1 mixture of 30% HNO_3 (Seastar) and 30% H_2O_2 (Seastar™) together with a pre-weighed highly pure ^{84}Sr spike. The samples decomposed typically within one hour, after which the solutions were dried down on a hotplate at 80 °C. Samples were taken up in a few drops of 3N HNO_3 and loaded on especially prepared disposable pipette-tip columns containing 0.2 ml, intensively pre-cleaned SrSpec™ (Eichrome Inc./Tristchem), mesh 50-100 resin. The elution recipe essentially follows that of Horwitz et al., [48].

Finally, thermal ionization mass spectrometry was used to determine the Sr isotope ratios. Samples were dissolved in 2.5 µl of a Ta_2O_5-H_3PO_4-HF activator solution and directly loaded onto previously outgassed 99.98% single rhenium filaments. Samples were measured at 1250-1300 °C in dynamic multi-collection mode on a VG Sector 54 IT mass spectrometer equipped with eight Faraday detectors (Institute of Geography and Geology, University of Copenhagen). Five nanogram loads of the NBS 987 Sr standard gave $^{87}Sr/^{86}Sr = 0.710236 \pm 0.000010$ (n=10, 2σ). Procedural blanks, with $^{87}Sr/^{86}Sr$ values ranging between 0.7085 to 0.7092, varied along with the different analytical procedures but remained below 150 pg. These contamination levels, when compared to the amounts of strontium deduced from the signal intensities during the analyses of the samples and

decontamination solutions, have no significant effects on the isotopic compositions of the samples analysed.

Extraction and sequencing of DNA from scalp hair

Circa 300 mg of hair was sampled from the Egtved Girl using sterilized equipment according to established standards [49]. Two DNA extractions were conducted, each on c.100 mg of hair. Following a brief wash in 5% hypochlorite (to minimize surface DNA contamination) and molecular grade H_2O, the DNA was extracted using phenol-chloroform combined with MinElute columns (Qiagen). The silica bound DNA was purified sequentially with AW1/AW2 wash buffers (Blood and Tissue Kit, Qiagen), Salton buffer (60% Guanidine Thiocyanate and 40% H_2O) and PE buffer, before being eluted in 60 µl EB buffer (both Qiagen). The extracts had a yellowish colour which indicated the presence of pigments, potentially inhibiting downstream amplification. Therefore, one of the extracts (MA218) was purified again with the same buffers as above, to obtain a cleaner extract, but one with a lower DNA concentration.

Following extraction, 20 µl of DNA extract was built into a blunt-end library using the NEBNext DNA Sample Prep Master Mix Set 2 (E6070) and Illumina specific adapters [50]. The libraries were prepared according to manufacturer's instructions, with a few modifications outlined below [51] . As ancient DNA is already highly fragmented, the initial nebulization step was skipped. The end-repair step was performed in 25 µl reactions using 20 µl of DNA extract. This was incubated for 20 minutes at 12°C and 15 minutes at 37°C, and purified using PN buffer with Qiagen MinElute spin columns, and eluted in 15 µl. Next, Illumina-specific adapters (prepared as in [51]) were ligated to the end-repaired DNA in 25 µl reactions. The reaction was incubated for 15 minutes at 20°C and purified with PB buffer on Qiagen MinElute columns, before eluted in 20 µl EB Buffer. The adapter fill-in reaction was performed in a final volume of 25 µl and incubated for 20 minutes at 37°C

followed by 20 minutes at 80°C to inactivate the Bst enzyme. The entire DNA library (25 µl) was then amplified and indexed in a 50 µl PCR reaction, mixing with 5 µl 10X PCR buffer, 4 µl MgCl2 (50 mM), 1 µl BSA (20 mg/ml), 0.5 µl dNTPs (25 mM), 1 µl of each primer (10 µM, inPE forward primer + indexed reverse primer), and 1 µl AmpliTaq Gold DNA Polymerase (Applied Biosystems). Thermocycling was carried out for 5 minutes at 94°C, followed by a variable number of cycles of 30s at 94°C, 30s at 60°C and 40s at 72°C, and a final 7- minute elongation step at 72°C. Due to the low DNA concentration in these two extracts, the libraries required a large number of PCR cycles (22 for MA219 and 32 for MA218) to produce a sequenceable product. The amplified library was purified with PB buffer on Qiagen MinElute columns, before being eluted in 30 µl EB.

The two DNA libraries were profiled on an Agilent Bioanalyzer 2100, pooled with other indexed libraries (different projects), and shot-gun sequenced (100 bp, single read) in two different sequencing runs on Illumina HiSeq 2000 platforms at the National High-throughput DNA Sequencing Centre, University of Copenhagen. The sequences were base called and sorted bioinformatically by index. Adapter sequences were trimmed off and reads shorter than 30 bp were removed using AdapterRemoval v.1.5.2 [31] . Mapping against the human reference genome (hg19) was conducted with BWA v. 0.7.5 [32] with seeding disabled (-l 1000). Duplicates were removed from the bam file using SAMtools v. 0.1.18 [33] and only reads with mapping quality ≥ 25 were retained.

<u>Stable isotopes of scalp hair</u>

Hair samples were prepared according to standard protocols [9] at the University of Bradford's stable Isotope Facility. Briefly, adherent soil and exogenous organic deposits were removed from the fibre surface by overnight soaking/ gentle agitation in organic solvent (2:1 methanol:chloroform), followed by sonication (3 x 15min). The organic solvent was then removed and the hair sample

itself rinsed in deionized water (x3 separate washes, each with sonication). The final wash was decanted off and the cleaned sample was then frozen, lyophilized and preconditioned for weighing into tin capsules. Fibres were carefully orientated and aligned relative to the proximal end and to one another, so that fibre segments could be weighed into tin capsules and analyzed for a diachronic picture of change.

Prepared hair samples were combusted in a Europa Scientific Geo 20/20 isotope ratio mass spectrometer coupled to a Roboprep elemental analyser. Isotopic concentrations for each element are expressed in relation to international standards so that the relative difference between the sample isotope ratio and that of the standard is expressed by use of the δ notation, with units expressed as per mil (‰). Carbon is measured relative to CO_2 prepared from a Cretaceous belemnite, from the Peedee Formation, South Carolina, whereas atmospheric N_2 is used as the standard for nitrogen. The carbon to nitrogen atomic ratio (C:N) offers a measure for purity of the hair samples, given that accepted values for modern hair are normally expected to fall within the range 3.0–3.9 [52].

Morphological investigations of scalp hair from the Egtved Girl

Selected human hairs from the Egtved Girl were mounted in permanent mounting medium (Safe-T-Mount; R.I. 1.52); all were mounted on conventional glass microscope slides and 0.17mm thick cover slips. Microscopical investigation was performed with an Olympus compound transmitted light microscope, equipped with objectives ranging from 40-400x magnification. Scale cast patterns and cross-sections were produced in accordance with Tridico et al., [53].

Microscopy of wool fibres

Microscopic investigation and measurements of the diameter of the individual wool fibres sampled from the textile yarns were performed with a Zeiss Primo Star iLed transmitted light microscope

with objectives ranging from 40-400x magnification, and images were captured with an AxioCam ERc5s digital camera. Fibre samples were mounted in liquid paraffin between conventional glass microscope slides and cover slips. A minimum of 100 fibres from each yarn sample were measured for their thicknesses on the photographs using the camera software (Supplementary Table S4). Data are depicted in a histogram using 2 µm thickness intervals (Supplementary Figure S6).

Supplementary Table S4. Summary of wool fibre thickness analyses.

Textile museum number	Textile	Yarn	Fibres measured	Magnification	Mode (µm)	Range (µm)	Skewness
B11836	Skirt	string	97	100x	16	10-24, 45	0.4
B11833	Blanket	light weft	118	100x	16	10-26, 29, 33, 63, 65, 109	0.6
B11834	Blouse	warp	266	40x	13	9-26, 37, 45, 61, 88	0.4
B11835	Belt	weft	339	40x	13	6-21, 23-24, 29, 39, 60, 101, 103, 119, 127, 149	0.4
B11833	Blanket	warp	120	100x	14	10-22, 24, 54, 60, 73, 102, 140, 144	0.6
B11833	Blanket	dark weft	117	100x	17	10-24, 37, 79, 84, 96	0.5
B11849	Bundle	warp/weft	116/143	100x	19,84/18,27	9-28, 30, 33, 59, 76, 94/10-25, 27-28, 30, 86, 124	0.53 / 0.36
B11847	Cord		120	100x	17	10-25, 27, 31, 40, 42, 44, 97-98	0.6

References:

34 AlQahtani, S. J., Hector, M. P. & Liversidge, H. M. Brief communication: The London atlas of human tooth development and eruption. *American Journal of Physical Anthropology* **142**, 481-490 (2010).

35 Harbeck, M. et al. Research potential and limitations of trace analyses of cremated remains. *Forensic Sci.Int.* **204**, 191-200 (2011).

36 Williams, L.J., White, C.D. & Longstaffe F.J, Improving stable isotopic interpretations made from human hair through reduction of growth cycle error. *American Journal of Physical Anthropology,* **145,** 125-136 (2011).

37 Wilson, A.S. & Gilbert, M.T.P., Hair and Nail in Thompson, T. & Black S. (eds) Forensic Human Identification: an Introduction, *CRC Press, Boca Raton*, 147-174 (2007).

38 Malaspinas, A.-S. et al. bammds: a tool for assessing the ancestry of low-depth whole-genome data using multidimensional scaling (MDS*). Bioinformatics* **30**, 2962-2964 (2014).

39 Thompson AH, Wilson AS & JR, E., Hair as a Geochemical Recorder: Ancient to Modern in Treatise on Geochemistry: Archaeology & Anthropology, 2nd Edn. (ed Cerling TE) **14,** 371-393, *Elsevier; Cambrigde* (2014).

40 Schoeninger, M. J. & DeNiro, M. J. Nitrogen and carbon isotopic composition of bone collagen from marine and terrestrial animals. *Geochim. Cosmochim. Acta* **48**, 625-639 (1984).

41 Bradfield, R.B. & Jelliffe, D.B. Hair-colour changes in kwashiorkor. *Lancet,* **16,** 461-2 (1974).

42 Melton, N., et. al., Gristhorpe Man: an Early Bronze Age log-coffin burial scientifically defined, *Antiquity* **84**, 796–815 (2010).

43 Seta, S., Sato, H., Miyake, B. in *Forensic Science Progress* **2** (ed A. Maehly, Williams, R.L.) 47-166 (Springer-Verlag, 1988).

44 Deedrick, D. W., Koch, S.L. Microscopy of Hair Part 1: A Practical Guide and Manual for Human Hairs. *Forensic Science Communications* **6** (2004).

45 Ryder, M. L. Sheep and Man. 856 (Gerald Duckworth & Co Ltd., 2007).

46 Rast-Eicher, A. & Bender Jørgensen, L. Sheep wool in Bronze Age and Iron Age Europe. *Journal of Archaeological Science* **40**, 1224-1241 (2013).

47 Ryder, M. L. A re-assessment of bronze age wool. *Journal of Archaeological Science* **10**, 327-331 (1983).

48 Horwitz, E. P., Chiarizia, R. & Dietz, R. W. A novel strontium-selective extraction chromatographic resin. *Solvent Extraction and Ion Exchange* **10**, 313-336 (1992).

49 Allentoft, M. E. Recovering samples for ancient DNA research -guidelines for the field archaeologist. *Antiquity* **87,** Issue 338 (2013).

50 Meyer, M. Kircher, M. Illumina sequencing library preparation for highly multiplexed target capture and sequencing. *Cold Spring Harbr Protocols* 6, pdb.prot5448 (2010).

51 Orlando, L. et al. Recalibrating Equus evolution using the genome sequence of an early Middle Pleistocene horse. *Nature* **499**, 74-78 (2013).

52 O'Connell, T. C. & Hedges, R. E. M. Isotopic comparison of hair and bone: Archaeological analyses. *Journal of Archaeological Science* **26**, 661-665 (1999).

53 Tridico, S. R., Rigby, P., Kirkbride, K. P., Haile, J. & Bunce, M. Megafaunal split ends: microscopical characterisation of hair structure and function in extinct woolly mammoth and woolly rhino. *Quaternary Science Reviews* **83**, 68-75 (2014).
